# First Draft Genome Sequence of a Member of the Genus *Planomicrobium*, Isolated from the Chandra River, India

**DOI:** 10.1128/genomeA.01259-13

**Published:** 2014-02-06

**Authors:** Richa Salwan, Mohit Kumar Swarnkar, Anil Kumar Singh, Ramesh Chand Kasana

**Affiliations:** a Hill Area Tea Science Division, CSIR–Institute of Himalayan Bioresource Technology, Palampur, India; b Division of Biotechnology, CSIR–Institute of Himalayan Bioresource Technology, Palampur, India

## Abstract

We report the first draft genome sequence of a member of the genus *Planomicrobium*, isolated from a soil sample from the Chandra River, located in the cold deserts of Himachal Pradesh, India. The draft genome assembly for *Planomicrobium glaciei* strain CHR43 has a size of 3,900,800 bp with a G+C content of 46.97%.

## GENOME ANNOUNCEMENT

The genus *Planomicrobium* is composed of aerobic, motile, Gram-positive to Gram-variable rod- to coccus-shaped bacterial strains. So far, nine species of the genus *Planomicrobium* have been defined, *viz*., *P. koreense*, *P. okeanokoites*, *P. mcmeekinii*, *P. alkanoclasticum*, *P. psychrophilum*, *P. chinense*. *P. glaciei*, *P. stackebrandtii*, and *P. flavidum*. Members of the genus *Planomicrobium* have been isolated from fermented seafood, marine mud, Antarctic samples, intertidal sediments, and glacier and marine solar salterns (1). However, a genome sequence for any species of the genus *Planomicrobium* had not yet been determined. The psychrotolerant bacterium *Planomicrobium glaciei* CHR43 was isolated from a soil sample from the Chandra River (latitude 32°24′55″, longitude 77°14′06″), located in a cold desert area of the Lahaul and Spiti district, Himachal Pradesh, India. The genome of *P. glaciei* CHR43 was sequenced, given the organism's ability to grow and produce protease enzyme over a wide range of temperatures (5 to 42°C). The whole-genome shotgun sequencing of *P. glaciei* CHR43 was performed using the Illumina Genome Analyzer IIx in the 76-bp paired-read format. A total of 73,533,050 raw reads were obtained with 5,588,511,800 bp of raw sequence. To obtain the high-quality (HQ) data, reads were filtered using NGS QC toolkit v 2.3 (2) (cutoff read length for HQ, 70%; cutoff quality score, 20). A total of 57,816,970 (78.63% raw reads) adaptor/primer free reads were used for *de novo* genome assembly using Abyss v 1.3.4 (3). In the data set, the k parameters (from 29 to 71) were optimized for best assembly, and at 68mers, 82% (23,717,433) of the reads were found to be paired and 14.7% (4,252,610) of the reads were singletons. The final assembly contains 103 contigs with a total size of 3,900,800 bp, an *N*_50_ contig length of 66,943 nucleotides (nt), the longest contig length of 442,487 nt, and a G+C content of 46.97%. The annotation was performed with the help of the RAST (Rapid Annotation using Subsystem Technology) server using the Glimmer 3 option (4), which predicted 3,934 coding genes, 76 RNA genes, and 406 predicted SEED subsystem features.

A comparison of the CHR43 genome sequence with existing genome sequences, performed using the RAST server, revealed that *Lysinibacillus sphaericus* C3-41 (score 521) and *Bacillus* sp. strain B-14905 (score 495) are its closest neighbors. The genome analysis of *P. glaciei* showed that all the genes for the Embden-Meyerhof pathway (glycolysis) were present. The presence of a gene for the enzyme methylglyoxal synthase, which allows the bacterium to bypass the lower part of glycolysis in carbon-rich but phosphorus-limited conditions, is an indication that *P. glaciei* prefers glycolytic substrates (5). *P. glaciei* also has all the enzymes for the pentose phosphate pathway. In *P. glaciei*, genes for enzymes for the utilization of fructose, galactose, mannitol, sucrose, ribose, trehalose, d-mannose, and lactose were also present. In addition, genes encoding peptidase/protease (93 genes), lipase (13 genes), and cold shock proteins (5 genes) were found.

### Nucleotide sequence accession numbers.

This whole-genome shotgun project has been deposited at DDBJ/EMBL/GenBank under the accession no. AUYR00000000. The version described in this paper is first version, AUYR01000000.

## References

[1] Jung YT Kang SJ Oh TK Yoon JH Kim BH 2009 *Planomicrobium flavidum* sp. nov., isolated from a marine solar saltern, and transfer of *Planococcus* stackebrandtii Mayilraj et al. 2005 to the genus *Planomicrobium stackebrandtii* comb. nov. Int. J. Syst. Evol. Microbiol. 59(Pt 12):2929–2933. 10.1099/ijs.0.009191-0 19628589

[2] Patel RK Jain M 2012 NGS QC Toolkit: a toolkit for quality control of next generation sequencing data. PLoS One 7:e30619. 10.1371/journal.pone.0030619 22312429 PMC3270013

[3] Simpson JT Wong K Jackman SD Schein JE Jones SJ Birol I 2009 ABySS: a parallel assembler for short read sequence data. Genome Res. 19:1117–1123. 10.1101/gr.089532.108 19251739 PMC2694472

[4] Aziz RK Bartels D Best AA DeJongh M Disz T Edwards RA Formsma K Gerdes S Glass EM Kubal M Meyer F Olsen GJ Olson R Osterman AL Overbeek RA McNeil LK Paarmann D Paczian T Parrello B Pusch GD Reich C Stevens R Vassieva O Vonstein V Wilke A Zagnitko O 2008 The RAST server: rapid annotations using subsystems technology. BMC Genomics 9:75. 10.1186/1471-2164-9-75 18261238 PMC2265698

[5] Rodrigues DF Ivanova N He Z Huebner M Zhou J Tiedje JM 2008 Architecture of thermal adaptation in an *Exiguobacterium sibiricum* strain isolated from 3 million year old permafrost: a genome and transcriptome approach. BMC Genomics 9:547. 10.1186/1471-2164-9-547 19019206 PMC2615787

